# Nonreproductive effects are more important than reproductive effects in a host feeding parasitoid

**DOI:** 10.1038/s41598-022-15296-2

**Published:** 2022-07-06

**Authors:** Yibo Zhang, Xiaocao Tian, Hao Wang, Cristina Castañé, Judit Arnó, Suran Wu, Xiaoqing Xian, Wanxue Liu, Nicolas Desneux, Fanghao Wan, Guifen Zhang

**Affiliations:** 1 State Key Laboratory for Biology of Plant Diseases and Insect Pests, Institute of Plant Protection, Chinese Academy of Agricultural Sciences, Beijing, 100193 China; 2 College of Plant Health & Medicine, Qingdao Agricultural University, Qingdao, 266109 China; 3 Sustainable Plant Protection Department, Institute for Research and Technology in Agriculture (IRTA), 08348 Cabrils, Barcelona, Spain; 4 Institute of Tropical Bioscience and Biotechnology, Chinese Academy of Tropical Agricultural Sciences, Haikou, China; 5 INRAE, CNRS, UMR ISA, Université Côte d’Azur, 06000 Nice, France; 6 Scientific Observing and Experimental Station of Crop Pests in Guilin, Ministry of Agriculture, Guilin, China

**Keywords:** Agroecology, Behavioural ecology

## Abstract

When female host feeding parasitoids encounter a potential host, they face a complicated trade-off between either laying an egg for investing in current reproduction or feeding on or killing the host for future reproduction. Few studies have measured these behavioral shift patterns in a given host-parasitoid association thus far. We systematically assessed the behavioral shifts and life history traits of a host feeding parasitoid, *Necremnus tutae,* on different instars of its host *Tuta absoluta. N. tutae* females, as idiobiont host feeding parasitoids, can act on the 1st–4th instar larvae of *T. absoluta* by either host feeding, parasitizing or host killing. Moreover, a significant behavioral shift was observed on different instar hosts. *N. tutae* preferred to feed on the young hosts (1st and 2nd instars), lay eggs on middle-aged hosts (3rd instars) and kill old hosts (4th instars) by ovipositor-mediated stinging. The offspring of *N. tutae* showed a significant female-biased sex ratio, with the number of instars of *T. absoluta* larvae that were parasitized increasing. Specifically, nonreproductive host mortality induced by host feeding and host killing accounted for high percentages of the total mortality (ranging from 70% on 3rd instar hosts to 88% on 1st instar and 4th instar hosts). We hypothesize that *N. tutae* could be not merely a parasitoid but also a predator. Our results shed light on the nonreproductive abilities of a host feeding parasitoid that should be given further attention, especially when evaluating the efficacy of parasitoids.

## Introduction

Insect parasitoids are important components of natural communities and are used to control insect pests in biological control programs worldwide^1^. In their interactions with hosts, parasitoids kill the hosts either by laying eggs in/upon hosts followed by offspring development (i.e., reproductive mortality) or by directly feeding on the hosts (host feeding, only exhibited by host feeding parasitoids)^2^. Host killing (or host stinging) behavior, in which a parasitoid can directly kill hosts with its ovipositor without reproductive behavior, has been observed in many parasitoids^3–5^. As parasitoids are very small in size, it is time consuming and labor intensive to distinguish their host killing behavior from other action modes^4,5^. Hence, for host feeding parasitoids, host killing was erroneously attributed to host feeding or was completely overlooked by many previous studies, resulting in the underestimation of their biological control efficacy^6,7^. That is, host killing, as a type of nonreproductive behavior, constitutes a hidden dimension of host-parasitoid trophic networks. To date, few studies have measured the three action modes (parasitism, host feeding and host killing) in a given host-parasitoid association^5^.

When female parasitoids encounter a host, they may first insert their ovipositor into the host to assess the suitability of the host^8^. Thereafter, the female parasitoids can either lay an egg and thus invest in current reproduction or feed on the host and thus invest in future reproduction^2^. During this process, they face a trade-off between current reproduction and future reproduction depending on internal factors (female physiological stage) and external factors (e.g., host development stage, host density)^2,9^. Female parasitoids give preference to host feeding when they are young in order to develop more eggs, as they usually harbor a low egg load and stay in a low-nutrition state^10^. For example, *Aphytis melinus* DeBach (Hymenoptera: Aphelinidae) females invest more in host feeding than in oviposition when they have a lower egg load and fewer nutritional reserves^11^. With regard to external factors, female parasitoids prefer to feed on hosts of poor quality for their nutrition while saving hosts of higher quality for oviposition and offspring development^12,13^. In addition to host feeding and parasitic oviposition, some parasitoid species from holometabolous (Lepidoptera, Coleoptera, Diptera) and hemimetabolous (Hemiptera) host taxa are known to exhibit host killing behavior or pseudoparasitism^5^, in which parasitoids tend to reject the host without ovipositing or host feeding after stinging herbivore hosts with their ovipositor. However, the ecological significance of this behavior is not very clear. Therefore, it would be interesting to determine how a given parasitoid species shifts among parasitic oviposition, host feeding and host killing when confronted with host larvae at different developmental stages. Addressing this subject could enhance our understanding of host-parasitoid biological interactions.

*Tuta absoluta* (Meyrick) is native to Peru in western South America^14^. Before 2006, it was found only in South American countries and Easter Island^15,16^. However, after its introduction into Europe in 2006, it spread rapidly throughout Afro-Eurasia and has become a major threat to tomato production worldwide^17–21^. In 2017, *T. absoluta* was detected in Xinjiang, China^22^, and management methods are currently being developed^20,23,24^. It mainly affects the photosynthesis of plants by mining and feeding on the leaves of the host plants during its larval stage^25^, but it also eats the stems and fruits of plants at high population densities, causing plant death and fruit fall off and rot, resulting in substantial economic losses^15^. The areas that were invaded by *T. absoluta* early on (mainly in Europe) have reduced the proportion of chemical control and have shifted to an integrated pest management (IPM) system with biological control at its core^23^, mitigating the potential negative effects of pesticides on beneficial arthropods^26^. *Necremnus tutae* Ribes & Bernardo (Hymenoptera: Eulophidae), which is native to and widespread in Europe and Africa, is a typical idiobiont-synovigenic host feeding eulophid parasitoid, the female adults of which kill or paralyze the host by injecting venom before laying eggs and feeding on the host^27^. This species has been identified as a promising biological control agent for *T. absoluta*^17,28–30^, as it not only parasitizes the larvae^31^ but also can directly feed on and/or kill all instars of *T. absoluta* larvae^30^. Moreover, for this organism, hosts killed by host killing can be easily distinguished from those killed by host feeding and parasitism^30^. Hence, *N. tutae* could be a good candidate for investigating the behavioral shifts among parasitism, host feeding and host killing when the parasitoid encounters hosts at different developmental stages.

To deepen our understanding of host-parasitoid biological interactions and to explore whether parasitoids can choose different attacking behaviors and exhibit different life history traits when they meet different instar hosts, this study assessed the host preference and life history traits of a host feeding parasitoid, *N. tutae,* in response to different instars of *T. absoluta* larvae. We described the *N. tutae* behavioral shift from oviposition to host feeding and/or host killing and further compared the life history traits of adult parasitoids when they encountered different instar hosts under laboratory conditions.

## Results

### Life history traits and behavioral shift of N. tutae on different host instars

#### Life history traits

The number of hosts on which *N. tutae* females fed and their host feeding proportion were significantly different among the different host instars of *T. absoluta* offered (host feeding: *F*_3, 87_ = 14.75, *P* < 0.0001, host feeding proportion: *F*_3, 87_ = 85.43, *P* < 0.001, Table 1). Both host feeding level and host feeding proportion decreased with host instar stage. There was no significant difference in the number of hosts killed for the different instars of *T. absoluta* larvae offered (*F*_3, 87_ = 0.55, *P* = 0.651, Table 1). Both the parasitism level and parasitism proportion of *N. tutae* females were significantly different among the different host instars offered (parasitism: *F*_3, 87_ = 4.66, *P* = 0.005, parasitism proportion: *F*_3, 87_ = 17.08, *P* < 0.001, Table 1). The parasitoids laid most of their eggs on 3rd and 2nd instar larvae, an intermediate number of eggs on 1st instar larvae and the fewest number of eggs on 4th instar larvae.

**Table 1:** Life history traits of *Necremnus tutae* females when different instars of *Tuta absoluta* were offered.

Host instars offered (n)	Host feeding^a^	Host feeding proportion^b^	Egg laying^c^	Parasitism proportion^d^	Host killing^e^	Host killing proportion^f^	Total host mortality^g^	Longevity (days)
1st instar (26)	45.4 ± 5.8 a	0.62 ± 0.03 a	12.1 ± 3.3 ab	0.12 ± 0.02 b	23.4 ± 4.8	0.26 ± 0.02 c	80.9 ± 13.1 a	19.4 ± 2.3 a
2nd instar (24)	29.7 ± 4.9 b	0.41 ± 0.02 b	21.5 ± 5.7 a	0.25 ± 0.02 a	25.1 ± 5.0	0.34 ± 0.02 bc	76.2 ± 14.9 ab	15.9 ± 2.2 a
3rd instar (20)	27.0 ± 2.8 b	0.33 ± 0.01 c	25.5 ± 3.9 a	0.30 ± 0.02 a	31.0 ± 3.8	0.37 ± 0.02 b	83.4 ± 9.7 a	12.5 ± 1.2 ab
4th instar (21)	4.5 ± 0.9 c	0.13 ± 0.02 d	5.8 ± 1.6 b	0.12 ± 0.02 b	23.9 ± 3.9	0.75 ± 0.03 a	34.2 ± 6.0 b	7.0 ± 0.9 b

^a^No. of hosts fed upon.

^b^Host feeding/total host mortality.

^c^No. of hosts parasitized.

^d^Egg laying/total host mortality.

^e^No. of hosts stung without feeding upon them.

^f^Host killing/total host mortality.

^g^Total no. of dead hosts.

The total mortality of host larvae was significantly different among the *T. absoluta* instars offered (*F*_3, 87_ = 3.51, *P* = 0.018, Table 1), and higher mortality was observed in 1st and 2nd instar larvae than in 4th instar larvae, with intermediate values for 3rd instar larvae. Nonreproductive mortality (host feeding + host killing) caused by the parasitoid accounted for a very high proportion of the total host mortality (range 69% on 3rd instar larvae to 85% on 1st instar larvae).

The longevity of *N. tutae* females was significantly different when they were fed upon distinct host instars (*F*_3, 87_ = 7.78, *P* < 0.001, Table 1), with a higher longevity observed when 1st and 2nd instar larvae were offered than when larvae of 4th instar were provided, with intermediate values for 3rd instar larvae.

#### Behavioral shift

**Figure 1:**
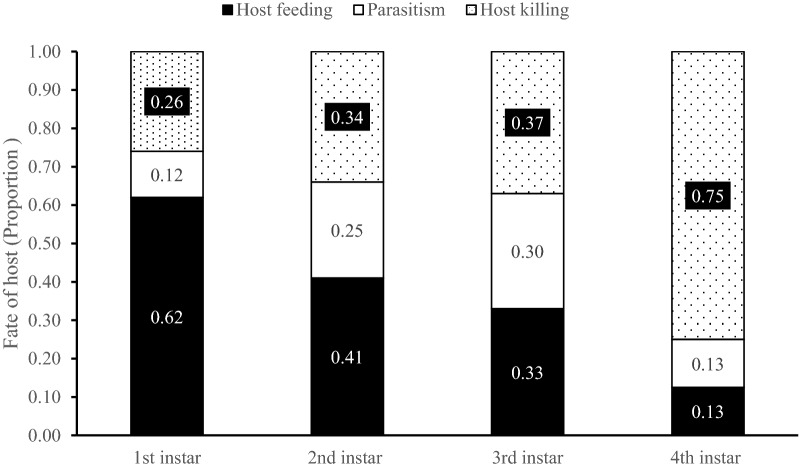
Behavioral shift among host feeding, parasitism and host killing of *Necremnus tutae* females when different instars of *Tuta absoluta* larvae were offered*.* The numbers in the bars indicate the proportion of the number of hosts killed by each behavior (host feeding, parasitism and host killing) in total host mortality.

**Figure 2:**
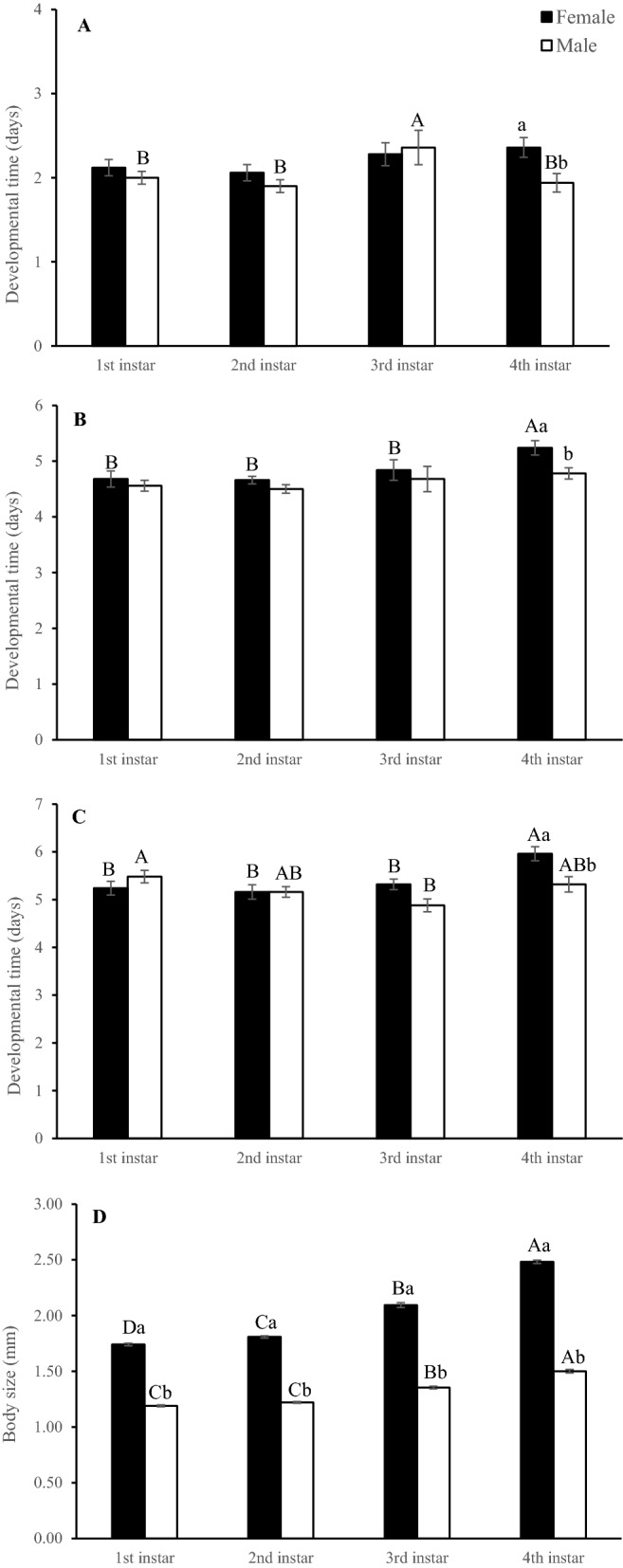
Mean developmental time (mean ± SE) of the egg stage (**A**), larval stage (**B**), and pupal stage (**C**) and body size (**D**) of female and male *Necremnus tutae* offspring on different instars of *Tuta absoluta* larvae. Bars topped by different capital letters within the same sex indicate a significant difference between different host instars; the different lowercase letters indicate that there was a significant difference in the same instar between female and male *T. absoluta* hosts; no lettering indicates no significant difference.

Parasitoids preferred to feed on 1st instar (*G* = 553.004, *df* = 2, *P* < 0.001, Fig. 1) and 2nd instar hosts (*G* = 31.828, *df* = 2, *P* < 0.001, respectively), while they preferred to parasitize 3rd instar larvae (*G* = 11.438, *df* = 2, *P* = 0.003) and kill 4th instar larvae (*G* = 400.254, *df* = 2, *P* < 0.001, Fig. 1).

### Development times of immature stages of N. tutae and body sizes of adults that developed on different instar larvae of T. absoluta

Both host instar offered and sex of offspring had significant effects on the development times of the egg, larval and pupal stages (Table 2, Fig. 2A, B, C), but the interaction between the two factors affected only the pupal stage (Table 2). The development time of the different developmental stages (egg, larval and pupal stages) tended to be longest in the 4th instar hosts. The duration of the pupal stage also differed between the sexes, with males developing faster than females (Fig. 2C).

**Table 2:** ANOVA results of the developmental times of eggs, larvae, and pupae and body sizes of female and male offspring of *Necremnus tutae* on different instar hosts of *Tuta absoluta*.

Source	df	Mean square	*F*	*P*
**Egg stage**				
Host instar	3	1.064	2.92	0.035
Sex	1	1.201	3.29	0.071
Host instar × sex	3	0.527	1.45	0.230
Model	8	113.903	312.33	< 0.001
Error	192	0.364		
Total	200			
**Larval stage**				
Host instar	3	1.887	3.95	0.009
Sex	1	2.531	5.29	0.023
Host instar × sex	3	0.311	0.65	0.584
Model	8	563.423	1177.63	< 0.001
Error	192	0.478		
Total	200			
**Pupal stage**				
Host instar	3	2.965	6.33	0.001
Sex	1	2.205	4.7	0.031
Host instar × sex	3	2.018	4.31	0.006
Model	8	708.375	1511.2	< 0.001
Error	192	0.468		
Total	200			
**Body size**				
Host instar	3	2.869	614.33	< 0.001
Sex	1	25.532	5466.94	< 0.001
Host instar × sex	3	0.478	102.56	< 0.001
Model	8	5.082	1088.25	< 0.001
Error	192			
Total	200

Both host instar and sex of offspring had significant effects on the body size of female parasitoids, and there was an interaction between host instars and sex of offspring (Table 2, Fig. 2D). Body size increased with instar stage at parasitism, and this effect was stronger for the larger sex (i.e., females).

### Survival and sex ratio of N. tutae offspring reared on different host instars

The proportion of survival of immature parasitoids did not vary among different instar hosts (ranging from 0.51 to 0.57, *F*_3, 87_ = 0.62, *P* = 0.604). The offspring sex ratio showed significant differences among different instar hosts (*F*_3, 74_ = 4.99, *P* = 0.003). The highest sex ratio (number of males/total emerged adults) was observed in the 1st instar host (0.86 ± 0.03, n = 26), followed by the 2nd instar host (0.66 ± 0.05, n = 24) and 3rd instar host (0.67 ± 0.04, n = 20), and the lowest was generated in the 4th instar host (0.50 ± 0.09, n = 21).

## Discussion

In this study, we found that *N. tutae*, as a typical idiobiont host feeding parasitoid, can act on the 1st–4th instar larvae of *T. absoluta* by host feeding, parasitizing or host killing. Meanwhile, the behavior of parasitoids shifted from host feeding to killing with the advancement of the host larval stage. The parasitism proportions in the 2nd and 3rd host instars were optimal, but both were less than 0.30, indicating that the majority of attacked hosts did not survive but also did not develop into parasitoids.

*N. tutae* can feed on all instar hosts and prefers 1st–2nd instar hosts. This indicates that this parasitoid prefers to feed on younger hosts. This result was consistent with many previous studies focused on host feeding parasitoids, such as *Bracon nigricans* Szépligeti (Hymenoptera: Braconidae)^32^, *Eretmocerus mundus* Mercet (Hymenoptera: Aphelinidae)^33^ and *Eretmocerus hayati* Zolnerowich and Rose (Hymenoptera: Aphelinidae)^34^. Compared with older hosts, younger hosts have lower nutritional quality and show a weaker immune response to parasitism from parasitoids; hence, parasitoids may easily successfully forage and feed on younger hosts than on older hosts^2^.

Host feeding parasitoids have a constant need for potential hosts to balance the trade-off between producing offspring for current reproduction and feeding for future reproduction^35^, so they easily evolve the ability to make rapid behavioral decisions based on their physiological requirements and host quality assessment. On the one hand, the behavioral decisions of parasitoids could be directly driven by their physiological requirements with respect to egg maturation. As synovigenic parasitoids, newly emerged *N. tutae* females usually have few mature eggs, so most of their eggs are developed and matured by obtaining nutrients by feeding on hosts after emergence. That is why host feeding by *N. tutae* was observed more in young females than in older females. On the other hand, we propose that feeding on younger hosts could be a general trend for all host feeding parasitoids^2,9^, as younger hosts usually have smaller body sizes and lower mobility. In addition, it is worth noting that our results showed some differences from those of Calvo et al.^36^. They indicated that *Necremnus artynes* (Walker) (Hymenoptera: Eulophidae) preferred feeding on the 2nd instar host of *T. absoluta* rather than the 1st instar host. This discrepancy could result from the different experimental protocols. They put four instars (1st–4th instar) in the same Petri dishes and simultaneously provided them to the parasitoid; moreover, this experiment was conducted within only 48 h. We hypothesize that access to mixed instar hosts could increase the difficulty for parasitoids in identifying 1st instar hosts for host feeding during such a short observation period, as 1st instar hosts are much smaller than 2nd instar hosts.

In the present study, *N. tutae* parasitized all instars of *T. absoluta* but preferred 2nd- and 3rd-instar hosts. The 1st instar host, which was the smallest in size, had less body fluid and resulted in smaller offspring. In contrast, 4th instar hosts had the largest body size with the highest amount of body fluid, but they had a strong capacity to resist the actions of the parasitoids. Based on our previous observation, 4th instar larvae of *T. absoluta* are able to escape the parasitoids by moving out of the leaf epidermis and quickly abandoning the current leaf upon encountering *N. tutae* that is beginning to probe the leaf^30^. Taken together, these two points indicate that the 2nd instar and 3rd instar hosts of *T. absoluta* are the optimal hosts for offspring production by *N. tutae*. Similar results have been reported for many other host feeding parasitoids, such as *Er. mundus*^33^, *E. hayati*^34^, *N. artynes*^36^.

In addition to host feeding and oviposition, host killing is also a very important measure for host control by some host feeding parasitoids^7^. However, entomologists have overlooked this behavior or directly combined it with host feeding, as host killing is very difficult to distinguish from host feeding^36,37^. In the present study, *N. tutae* showed a high capacity to kill all instar hosts by ovipositor-mediated stinging (ranging from 26 to 75% of offered hosts), exhibiting a preference for 4th instar hosts, wherein the proportion of total host mortality of 4th instar *T. absoluta* hosts caused by parasitoids reached up to 75%. In fact, the 4th instar host is used to escaping the attacks of parasitoids by moving out of the mine when it encounters a parasitoid^30^. During the escape process, parasitoids keep in step with the host and attempt to sting the host with their ovipositors. Once the host is successfully stung by the parasitoid, it experiences increasing paralysis until death as venom or another chemical substance is injected into the host body by the parasitoid^5,32,38^, even though it may still struggle to climb out of the mine and leave the current leaf. Because of its escape strategy, the 4th instar host is very difficult to parasitize, as the time window for oviposition in the host by the parasitoid is short, usually only 10 seconds^30^. Once the host leaves the mine, the parasitoid does not parasitize the host (personal observation by Yibo Zhang). According to this result, it may be more accurate to interpret host killing as unsuccessful parasitism killing the hosts. During the host killing process, the primary intention of the parasitoid could be parasitism by injecting venom while laying an egg. However, injecting venom can usually easily be completed, but laying eggs (parasitism) on later instar hosts may be interrupted by aggressive and agile behavior of the host, even if the host had been injected with venom by the parasitoid.

Besides, the high capacity of host killing of *N. tutae* and escaping behavior of *T. absoluta* could partly result from that *N. tutae* is not a natural parasitoid of *T. absoluta*, as the former is native to Europe/African regions but the later originated from South American. When an exotic invasive species possessing similar ecological and physiological attributes to indigenous hosts becomes abundant in the ecosystem, maladaptive oviposition decisions by parasitoids causing nonreproductive effects could intensify, leading to an evolutionary trap for indigenous parasitoids^39,40^. Similarly, the indigenous generalist egg parasitoid *Telenomus podisi* Ashmead (Hymenoptera: Scelionidae) accepts eggs of the newly invasive alien stink bug *Halyomorpha balys* (Stal) (Hemiptera: Pentatomidae) at high rates and causes some host eggs to abort development, but their offspring cannot successfully develop^39^. That is to say, native species may either evolve or learn mechanisms to cope with the invaders (e.g. through chemical defences, improved competitive abilities, predator-avoidance behavior) and ultimately persist on their own ^40^. From an evolutionary perspective, nonreproductive behavioral events (host killing) could create favorable conditions for the establishment of new, viable host-parasitoid associations.

A similar agility-based escape strategy of high-instar larvae that encounter parasitoids was recorded for another two host feeding larval parasitoids, *Stenomesius* sp. nr. *japonicus* (Ashmead) and *Necremnus* cf. *artynes* (Walker) (Hymenoptera: Eulophidae), in Spain by Chailleux et al.^37^. They observed host feeding and host stinging behaviors of two parasitoids in the laboratory but did not further assess the two behaviors and took them to be host killing without parasitism (90.2 larvae for *S. sp.nr. japonicus,* 26.2 larvae for *N. cf. artynes*). Van Driesche et al.^41^ also reported host killing behavior in two mealybug parasitoids, namely, *Epidinocarsis diversicornis* (Howard) and *Acerophagus coccois* Cox & Williams (Hymenoptra: Encyrtidae). In addition, some researchers have also described and assessed host stinging behavior in several host feeding parasitoids. Barret and Brunner^42^ indicated that the leaf miner parasitoid *Pnigalio flavipes* (Ashmead) (Hymenoptera: Eulophidae) exhibited three types of parasitoid-induced mortality (host stinging with oviposition, host feeding and host stinging without oviposition). Cebolla et al.^4^ confirmed that two host feeding parasitoids, *Aphtyis melinus* and *Aphtyis chrysomphali* (Mercet) (Hymenoptera: Aphelinidae), rejected approximately 30% of the California red scale *Aonidiella aurantia* (Maskell) (Hemiptera: Diaspididae) that it encountered. In addition, some researchers have also systemically investigated the host killing behavior of some host feeding parasitoids, such as *Encarsia tricolor* Forster (Hymenoptera: Aphelinidae)^43^, *B. nigricans*^32^ and *E. hayati*^34^. That is, host killing behavior by ovipositor stinging is as common as parasitism or host feeding in many host feeding parasitoids^5^. Even though some authors indicated that this behavior could be used to decrease pest density and consequently maintain plant quality for successful development of additional offspring^44^ or can provide a food reserve for the offspring via killing of more host larvae^45^, it is unfortunate that the mechanisms underlying this behavior and the decision-making process of the parasitoids are not clear to date.

The body size of the offspring of *N. tutae* increased with host instar age. We propose that this phenomenon could be attributed to the larger body size of older *T. absoluta* larvae compared to younger larvae. Generally, insects have to grow for a longer time to achieve a larger body size^46^. Obviously, there is a size development trade-off. The larvae develop faster in early instar hosts than in later instar hosts, at the cost of being smaller in body size in the former. Similar results were reported for *S. sp.nr. japonicus*^37^ and *B. nigricans*^47^. In addition, the sex ratio of the offspring of *N. tutae* showed a significant male bias as the host instar stage decreased. This result was consistent with many previous studies. Barret and Brunner^41^ found that female *P. flavipes* tended to emerge from the larger leaf miner stages, but males emerged from the smaller stages. Chailleux et al.^36^ indicated that the sex ratio of *S. sp.nr. japonicus* was female biased when hosts were older, and only females emerged when parasitoids developed in *T. absoluta* fourth-instar larvae. Similar results were also observed for *Bracon nigricans* in Europe^32^ and Africa^47^. That is, more male offspring of these parasitoids developed from younger hosts. This result was in accordance with males having a small time-budget advantage under sexual selection^48^, which means that smaller males may be more agile and maneuverable when courting and searching for mates, resulting in increased mating and reproduction success^49^. Meanwhile, smaller individuals require less food to support themselves, so smaller males should have free energy and time for any activity that increases their mating and reproductive success^50^. Based on our observations, male *N. tutae* actively seek, contact and mate with females during the mating process. On the other hand, female offspring generated larger body sizes on older instar hosts. This corresponds with fecundity selection in evolutionary theory^46^. This means fecundity generally increases with female size and may reach an asymptote at large body size, but fecundity selection favoring small female size has not been proposed or observed until now^51,52^. In summary, the male-biased sex ratio of *N. tutae* with variable instar hosts could result in optimal or better fitness and shed light on the sexual size dimorphism of *N. tutae*. This organism was under two major evolutionary forces: fecundity selection in females and sexual selection in males. However, the current results do not fully address this issue, and more research on *N. tutae* or other similar species needs to be conducted in the future.

A significant behavioral shift among host feeding, oviposition and host killing across different hosts in *N. tutae* was found in the present study. It preferred to feed on young hosts, lay eggs on middle-aged hosts and directly kill old hosts. These behavioral shifts could shed light on the mechanisms underlying the behavioral preference of host feeding parasitoids facing variable hosts. Meanwhile, as the nonreproductive mortality of *N. tutae* on its hosts accounted for a very high proportion of the total host mortality, up to 88% on 1st- and 4th-instar hosts, these results could broaden the current host-parasitoid population and community models by revealing a nonreproductive dimension (host killing) and could help to elucidate the potential of the nonreproductive effects to cascade through food chains and influence ecosystem services such as biological control. We propose that entomologists should give more attention to the nonreproductive abilities of host feeding parasitoids, and these should be considered when evaluating the efficacy of parasitoids as biological control agents.

## Methods

### *Tuta absoluta *and *Necremnus tutae* cultures

All plants and insects were maintained in a climate-controlled chamber (PGC-450, Xunon Instruments (Beijing) Co., Ltd., China) at 26 ± 1 °C with 70–80% relative humidity (RH) and a 14 h:10 h light:dark (L:D) photoperiod at the experimental station (Yuxi City, Yunnan Province, N24°21’, E102°32’, 6 m a.s.l.) of the Department of Biological Invasions (DBI), Institute of Plant Protection, Chinese Academy of Agricultural Sciences.

Approximately 80 pairs the tomato leaf miner, *Tuta absoluta,* were collected from tomato fields during the summer of 2019 in Yuxi city. A laboratory colony of *T. absoluta* was maintained in a gauze-covered cage (40 × 40 × 60 cm, mesh size = 120) on young tomato plants (pink fruit tomato, Shouyan PT326, Shandong Shouguang Vegetable-seed Industrial Group, China). When the six true leaves had fully developed, the tomato plants were transferred into gauze cages to maintain the colony of *T. absoluta*. To obtain a continuous supply of host larvae at the same age, two young tomato plants were infested daily with 40–50 adults in a gauze-covered cage (40 × 40 × 40 cm, mesh size = 120). After 24 h, all the adults were removed, and plants were checked daily until the offspring developed to the desired instar stage for the experiments. Based on our primary experiments, 1st instar larvae of *T. absoluta* usually appeared after days 4–5, 2nd instars on days 8–9, 3rd instars on days 11–12, and 4th instars on days 14–15.

In 2019, approximately 100 pairs of *Necremnus tutae* were collected from tomato fields close to the Institute for Research and Technology in Agriculture (IRTA) in Cabrils, Barcelona (Spain) (N41°30′, E2°22′, 6 m a.s.l.) and introduced to China. Since then, an indoor colony of *N. tutae* has been maintained on 2nd- to 3rd-instar larvae of *T. absoluta* on young tomato plants in the DBI laboratory*.* To avoid the risk of this parasitoid parasitizing other native species in China, the colony of *N. tutae* was reared in the greenhouse of the quarantine center of the DBI in Yunnan before assessing the risk posed by this parasitoid to other similar native species.

### Experimental setup

To obtain newly emerged parasitoids, *N. tutae* pupae were isolated in 1.5 ml tubes. When adult females emerged (less than 24 h old), males were introduced into the same tube, and after mating was observed (usually within a few minutes), they were considered mated young females. Then, each sample was transferred into a 10 ml tube containing filter paper slightly moistened with distilled water to provide water.

#### Behavioral preference and life history traits of N. tutae on different host instars

**Figure 3:**
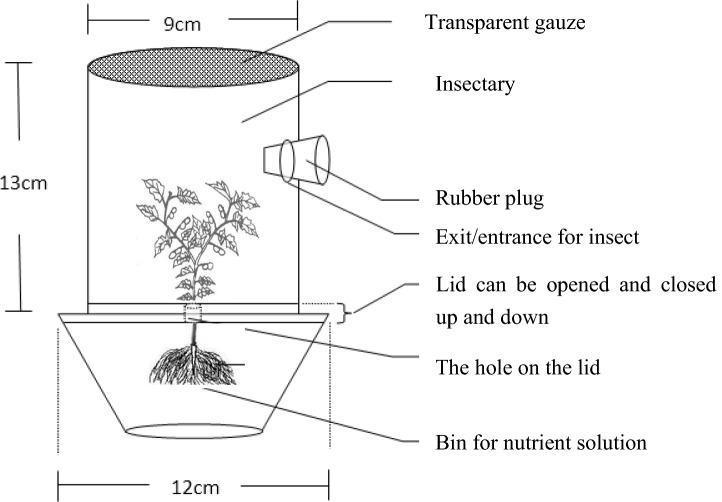
Microcosm for host suitability experiment of *Necremnus tutae* on *Tuta absoluta.*

To investigate the *behavioral* preference of *N. tutae* for different host instars, we designed a special cage (Fig. 3). The upper part was a cylindrical transparent plastic container (13 cm in height, 9 cm in diameter) with a gauze screen on top for ventilation, a lateral hole (1 cm in diameter) with a cotton plug and a bottom lid with a hole (1 cm in diameter) in the center. The lower part was another cylindrical transparent plastic container (6 cm in height, 15 cm in diameter) with a lid on top that also had a circular hole (1 cm in diameter). The two lids of the upper and lower containers were glued, keeping the holes of the lids aligned. The upper container was used to hold the tomato stalks infested with the desired host instar, and the lower container was used for watering the plant (see picture in Fig. 3). During this experiment, we did not use intact plants, as intact plants are too tall and take up more space; thus, we could not observe the exact natural behavior of the parasitoids.

After the tomato stalks infested with the desired instar larvae of *T. absoluta* in the tomato leaf (no less than 20 hosts) were moved into the special cage (Fig. 3), a newly emerged mated female was transferred into the upper part through the lateral hole. After 48 h, the female parasitoid was transferred into another cage with a tomato stalk until death. The stems and tomato leaves that were replaced were transferred into a Petri dish (9 cm in diameter) that had a fine layer of agar solution (5% w/v) to keep the leaves hydrated. The dishes were covered with a transparent plastic film with several small holes made with an insect pin for ventilation. After 96 h, we recorded the number of hosts that were fed upon, killed or parasitized. If the host larvae were parasitized, an egg or a small parasitoid larva (or possibly 2 or 3, but very rarely) could be easily found on or near the host; if the host was directly fed upon, the color of the host body became black or brown, and the body was flat and desiccated; if the host was killed, the color of the host body also became black or brown, but the body was full, and no eggs or larvae of parasitoids could be found^22^. The replication number of each host instar was no less than 20. The longevity of the female was also recorded.

#### Immature development, body size, survival and sex ratio of N. tutae offspring on different host instars

Parasitized larvae were randomly numbered to monitor the developmental times of the egg, larval and pupal stages of the parasitoid. Each Petri dish was checked 4 times daily from 8:00 to 20:00 (interval 3 h). The times of egg hatching, pupation and emergence of parasitoid offspring were recorded. The body size (dorsal length from the top of head to the end of the abdomen) of emerged adults was measured with a micrometer under a binocular microscope (SZ-61, Olympus, Japan). The total immature development time was the sum of the durations of the egg, larval and pupal stages. The number of replicates used to determine the developmental times of immature parasitoids on 1st, 2nd, 3rd and 4th instar larvae of *T. absoluta* was 50 (25 for females), 50 (25 for females), 50 (25 for females) and 50 (25 for females), respectively. Upon emergence, the total number of emerged parasitoids and sex ratio (number of males/total emerged adults) were calculated.

### Statistical analysis

To explore differences in life history traits (host feeding, host killing, total host mortality, fecundity, longevity, host feeding rate, parasitization rate, host killing rate, eclosion rate and the ratio of adult males to total offspring) of *N. tutae* females in response to *T. absoluta* larval instars, we compared the differences in each life history trait among different host instars by one-way ANOVA followed by Tukey’s honestly significant difference (HSD). The host feeding rate, parasitism rate and host killing rate of adult parasitoids and the eclosion rate and sex ratio of offspring were transformed by ARCSIN before analysis.

To explore whether the behavioral shifts among host feeding, oviposition and host killing of *N. tutae* in each host instar were significantly greater than expected, the G-test for goodness-of-fit was used. If no behavioral shift existed, the behavioral rate observed was equal to the expected rate on each instar host (host feeding:oviposition:host killing = 1:1:1).

To explore differences in the development times of eggs, larvae, and pupae and the body size of *N. tutae* offspring among the four host instars, a general linear model (GLM, two-way ANOVA) was used. Host instars (1st, 2nd, 3rd and 4th) and sex of offspring were the two factors, as the developmental times of each stage of female and male parasitoids met assumptions of normality and homoscedasticity. Tukey multiple comparisons between least square means were conducted when the interaction of two factors in the models was significant. If the interaction was not significant, one-way ANOVA complemented with the HSD test was further used to compare the difference in development times among different main factors (host instar and sex of offspring).

All of the analyses were conducted using SAS software (version 9.20).

### Ethics approval

This article does not contain any studies involving human participants or animals performed by any of the authors. All local, national or international guidelines and legislation were adhered to for the use of plants in this study.

### Consent for publication

Consent for publication was obtained from all authors included in the study.

## Data Availability

The dataset generated during the current study is available from the corresponding author upon request.
